# Acid-Denatured Green Fluorescent Protein (GFP) as Model Substrate to Study the Chaperone Activity of Protein Disulfide Isomerase

**DOI:** 10.3390/ijms12074625

**Published:** 2011-07-18

**Authors:** Rosa E. Mares, Samuel G. Meléndez-López, Marco A. Ramos

**Affiliations:** Facultad de Ciencias Químicas e Ingeniería, Universidad Autónoma de Baja California, Calzada Universidad 14418, Parque Industrial Internacional, Tijuana, Baja California 22390, México; E-Mails: rmares@uabc.edu.mx (R.E.M.); samuelmelendez@uabc.edu.mx (S.G.M.-L.)

**Keywords:** green fluorescent protein, protein disulfide isomerase, folding, chaperone

## Abstract

Green fluorescent protein (GFP) has been widely used in several molecular and cellular biology applications, since it is remarkably stable *in vitro* and *in vivo*. Interestingly, native GFP is resistant to the most common chemical denaturants; however, a low fluorescence signal has been observed after acid-induced denaturation. Furthermore, this acid-denatured GFP has been used as substrate in studies of the folding activity of some bacterial chaperones and other chaperone-like molecules. Protein disulfide isomerase enzymes, a family of eukaryotic oxidoreductases that catalyze the oxidation and isomerization of disulfide bonds in nascent polypeptides, play a key role in protein folding and it could display chaperone activity. However, contrasting results have been reported using different proteins as model substrates. Here, we report the further application of GFP as a model substrate to study the chaperone activity of protein disulfide isomerase (PDI) enzymes. Since refolding of acid-denatured GFP can be easily and directly monitored, a simple micro-assay was used to study the effect of the molecular participants in protein refolding assisted by PDI. Additionally, the effect of a well-known inhibitor of PDI chaperone activity was also analyzed. Because of the diversity their functional activities, PDI enzymes are potentially interesting drug targets. Since PDI may be implicated in the protection of cells against ER stress, including cancer cells, inhibitors of PDI might be able to enhance the efficacy of cancer chemotherapy; furthermore, it has been demonstrated that blocking the reductive cleavage of disulfide bonds of proteins associated with the cell surface markedly reduces the infectivity of the human immunodeficiency virus. Although several high-throughput screening (HTS) assays to test PDI reductase activity have been described, we report here a novel and simple micro-assay to test the chaperone activity of PDI enzymes, which is amenable for HTS of PDI inhibitors.

## 1. Introduction

Green fluorescent protein (GFP) is an autofluorescent protein that was first identified and isolated from the jellyfish, *Aequorea victoria* [1]. GFP is a soluble protein that contains a fluorophore consisting of three post-translationally modified amino acid residues (Ser^65^–Tyr^66^–Gly^67^) [2]. Because its fluorescence is remarkably stable, *in vitro* and *in vivo*, GFP has been widely used in several molecular and cellular biology applications [3,4]. Interestingly, GFP is resistant to denaturation by 8 M urea, 6 M guanidine hydrochloride, and 1% sodium dodecyl sulfate (SDS) [5]; moreover, it is unusually thermo-stable at different pH values and after chemical denaturation [6,7]. However, a low fluorescence signal has been observed after chemical-induced denaturation at pH 6.5 [8] and after acid-induced denaturation at pH 1.5 [9]. Acid-denatured GFP has been used as model substrate to study the protein folding activity of bacterial chaperones [10–13] as well as the chaperone-like activity of imidazole [14], and CPH nanogel [15].

Protein disulfide isomerase enzymes (PDI, EC 5.3.4.1) are eukaryotic oxidoreductases that catalyze the oxidation and isomerization of disulfide bonds in nascent polypeptides. PDI enzymes play a key role in the folding of proteins delivered to the secretory pathway; moreover, they are multifunctional proteins that display chaperone activity [16,17]. Interestingly, their function as oxidoreductase or chaperone is substrate-dependent [18–20]. Moreover, it seems that the chaperone activity of PDI might be independent of its enzymatic activity [21].

One function of chaperones is to suppress aggregation of non-native forms of proteins during the refolding or unfolding processes; however, *bona fide* chaperones are proteins that promote the complete folding of denatured substrates to their native conformation and are different from chaperone-like proteins which, on the contrary, only retain partially that ability (e.g., prevent aggregation) [22]. Here, we report the further application of GFP as a model substrate to study the chaperone activity of yeast and human PDI homologues (YPDI and HuPDI, respectively). Since refolding of acid-denatured GFP can be easily and directly monitored by real-time fluorescence, a simple micro-assay was used to study the effect of the chaperone or substrate concentration on protein folding kinetics. Moreover, this micro-assay format was used to evaluate the effect of a well-known inhibitor of PDI enzymes.

## 2. Results and Discussion

### 2.1. Acid-Denatured GFP as Model Substrate

**Figure 1:**
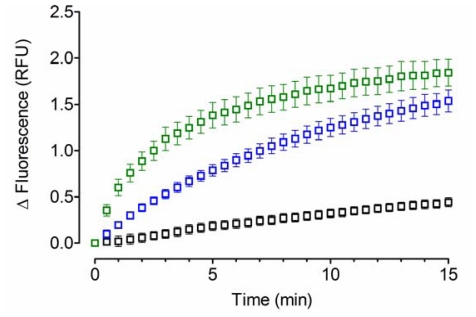
Green fluorescent protein **(**GFP) refolding kinetics. 0.05 μM of acid-denatured GFP was refolded in renaturing buffer containing 1 μM of a protein disulfide isomerase (PDI as chaperone): YPDI [blue] and HuPDI [green]; or in the absence of any chaperone [black]. Data represent mean ± S.E.M. (bars) of three independent experiments.

To demonstrate the feasibility of applying the acid-denatured GFP as substrate to study the chaperone activity of YPDI and HuPDI, a preliminary assay was performed (Figure 1). As expected, spontaneous refolding was observed in the absence of a molecular chaperone, with kinetics that followed an exponential one-phase association model with a rate constant (*k*) of 0.037 ± 0.021 min^−1^ and fluorescence at infinite time (*Plateau*) of 1.04 ± 0.48 RFU. Significant improvement of refolding was obtained with the assistance of YPDI or HuPDI (*p* < 0.05), indicating the participation of their chaperone activity. Furthermore, the refolding kinetics also followed an exponential one-phase association model with *k* values of 0.107 ± 0.012 min^−1^ for YPDI and 0.311 ± 0.027 min^−1^ for HuPDI, but with similar *Plateau* values (1.91 ± 0.12 and 1.78 ± 0.04 RFU, respectively). Interestingly, the *k* value exhibited by HuPDI was three-fold higher than that for YPDI. This dissimilarity could be likely related to the molecular differences in their substrate binding sites [23–25] and the multifunctional behavior of HuPDI [16,17].

### 2.2. Effect of PDI Concentration

**Figure 2:**
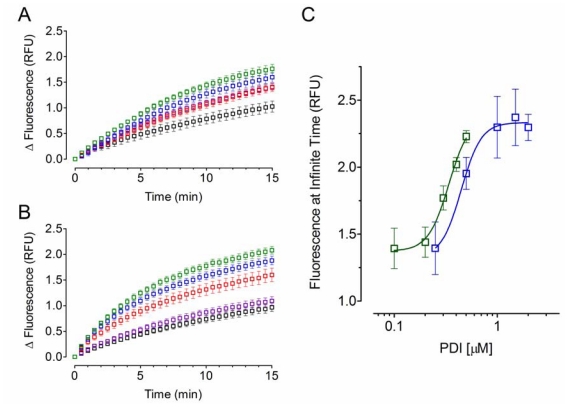
Effect of PDI concentration on its chaperone activity. 0.05 of acid-denatured GFP was refolded in renaturing buffer containing different μM concentrations of YPDI: 0 [black], 0.5 [purple], 1.0 [red], 1.5 [blue], and 2.0 [green] (**A**); or HuPDI: 0.1 [black], 0.2 [purple], 0.3 [red], 0.4 [blue], and 0.5 [green] (**B**); dependence of the refolding kinetics (fluorescence at infinite time, *Plateau*) on PDI concentration: YPDI [blue] or HuPDI [green] **(C)**. Data represent mean ± S.E.M. (bars) of three independent experiments.

Since acid-denatured GFP was a feasible substrate model and considering that refolding assisted by PDI proteins followed an exponential one-phase association model, the effect of PDI concentration on their chaperone activity was then analyzed. As observed in Figure 2, an amplification of the fluorescent signal over time was detected as result of increasing the PDI concentration (Figure 2A and 2B). Although, no significant change was observed in the *k* values (*p* > 0.05), the *Plateau* values were used to estimate the half maximal effective concentration (EC_50_) for each PDI protein (Figure 2C). The concentration/*Plateau* data were fitted to a four-parameter dose-response variable slope model and the apparent EC_50_ values of 0.45 ± 0.09 μM for YPDI and 0.34 ± 0.04 μM for HuPDI were obtained. Considering that there might be differences in the substrate affinity and specificity, the observed EC_50_ values suggest that both PDI proteins have similar chaperone activity.

### 2.3. Effect of Acid-Denatured GFP Concentration

**Figure 3:**
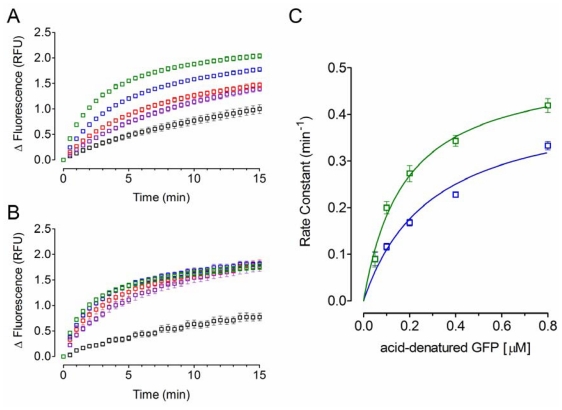
The effect of acid-denatured GFP concentration on PDI chaperone activity. Different μM concentrations of acid-denatured GFP were refolded in renaturing buffer containing 0.25 μM of YPDI (**A**) or 0.20 μM of HuPDI (**B**): 0.05 [black], 0.1 [purple], 0.2 [red], 0.4 [blue], and 0.8 [green]; dependence of the refolding kinetics (rate constant, *k*) on the substrate concentration: YPDI [blue] or HuPDI [green] (**C**). Data represent mean ± S.E.M. (bars) of three independent experiments.

Additionally, the effect of the substrate concentration on the chaperone activity of PDI was also determined. As shown in Figure 3, a higher fluorescence signal over time was detected as a consequence of increasing the concentration acid-denatured GFP (Figures 3A and 3B); thus, increasing *k* values were observed (Figure 3C), demonstrating that the chaperone activity PDI was dependent on the substrate concentration. Furthermore, the displayed increase on the *k* values was statistically significant (*p* < 0.05). Moreover, analysis of the concentration/*k* data with the Hill equation yields a *Hill* coefficient of 0.89 ± 0.11 and 1.06 ± 0.08 for YPDI and HuPDI, respectively, indicating that both chaperones bind one molecule substrate per monomer with no cooperativity. Thus, by fitting data to a specific one-binding site model, apparent *K_d_* values of 0.30 ± 0.09 μM for YPDI and 0.18 ± 0.03 μM for HuPDI were observed. These results add further evidence to the notion that differences in the substrate affinity and specificity exist [23–25]. Table 1 summarizes the kinetic parameters exhibited by the chaperone activity of YPDI and HuPDI.

### 2.4. Bacitracin Inhibits the Chaperone Activity of PDI

**Figure 4:**
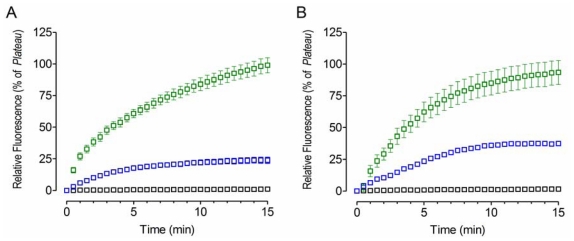
Effect of bacitracin on the PDI chaperone activity. 0.05 μM of acid-denatured GFP was refolded in renaturing buffer containing 2 μM of YPDI (**A**); or HuPDI (**B**), inhibited with 0 [green], 0.1 [blue], and 1 mM [black] of the antibiotic bacitracin. Data represent mean ± S.E.M. (bars) of three independent experiments.

The antibiotic bacitracin, a well-known inhibitor of PDI proteins [26], was then used to test the effect of an inhibitor on the chaperone activity of PDI (Figure 4). Typical inhibition kinetics was displayed, *i.e.*, diminished refolding was observed as the outcome of bacitracin binding to YPDI or HuPDI (Figures 4A and 4B), indicating the inhibition of its chaperone activity (*p* < 0.05). Interestingly, full inhibition was observed at 1 mM of bacitracin. Although, the apparent *k* values determined from the residual chaperone activity at 0.1 mM of bacitracin remain practically unchanged (*p* < 0.05), a reduction of the apparent *Plateau* was detected, 75% for YPDI and 56% for HuPDI. These results support the notion that bacitracin inhibits PDI apparently through competition for the substrate binding site [27].

## 3. Experimental Section

### 3.1. Vectors, Bacterial Strains, Enzymes and Chemicals

The *E. coli* XL1-Blue MRF’ strain and the plasmid pBluescript SK(-) were from Stratagene. The plasmid pQE30 and the Ni-NTA-agarose resin were from Qiagen. The plasmids pQBI25 and pCMV6-XL4/NM_000918.2 were from Qbiogene and Origene, respectively. The plasmid pCT38 was kindly provided by Christine Tachibana (University of Washington, USA) [28]. The routine molecular biology enzymes were from New England Biolabs. The biochemicals and other reagents were from Sigma-Aldrich.

### 3.2. Construction of Recombinant Plasmids

The jellyfish GFP gene (*sg*GFP, that is an optimized variant of the *A. victoria* GFP: F64L, S65C, I167T, in which solubility and protein folding has been improved for more rapid and efficient fluorophore formation) was obtained by endonuclease cleavage from plasmid pQBI25. A *Ksp*I-*Xba*I GFP-containing fragment was subcloned into the plasmid pBluescript SK(-). After that, a stretch of the multiple cloning sites, from *Bam*HI to *Eco*RI, was removed by endonuclease digestion, overhangs filling and autoligation. Then, a *Sac*I-*Hin*dIII GFP-containing fragment was subcloned into the plasmid pQE30. To get the recombinant plasmid pQHGF301, a segment from *Bss*HII to *Bam*HI was removed as previously. The PDI genes from yeast (YPDI) and human (HuPDI) were amplified by PCR using the plasmids pCT38 and pCMV6-XL4/NM_000918.2 as templates, respectively, and the corresponding synthetic oligonucleotides as primers (Table 2). Then, the amplified YPDI and HuPDI products were digested with specific endonucleases and subcloned into the plasmid pQE30, to get the recombinant plasmids pQYPDI and pQHuPDI. All recombinant plasmids were isolated from stable transformants and confirmed by endonuclease restriction analyses and DNA sequencing.

### 3.3. Expression and Purification of GFP, YPDI and HuPDI

The expression and purification of recombinant GFP, YPDI, and HuPDI proteins from *E. coli* XL1-Blue MRF’ cell cultures (harboring the plasmid pQHGF301, pQYPDI or pQHuPDI) was performed according to standard protocols.

#### GFP, YPDI and HuPDI expression

A single clone of each transformant was selected and grown overnight at 37 °C with agitation; the culture was diluted 100-fold in fresh media and grown at 37 °C for 2 h with agitation (pre-induction); then, IPTG was added to a final concentration of 0.1 mM and grown at 37 °C for another 4 h with agitation (protein induction). The cells were harvested by centrifuging at 9,500 rpm for 5 min.

#### GFP purification

The cell pellet was resuspended in 5 volumes of lysis buffer (8 M urea, 50 mM Tris-HCl, pH 8.0) and disrupted by vortexing (1–2 min). The total cell lysate was clarified by centrifugation at 9,500 rpm for 15 min (4 °C). Recombinant GFP was purified by Ni-chelating affinity chromatography following the native conditions protocol, described in *the QIAexpressionist*^®^ *manual* (Qiagen), to eliminate the denaturant and allow the on-column refolding. Finally, refolded GFP was collected from Sephadex™ G-25 columns (PD-10, Amersham Biosciences) using 20 mM Tris-HCl (pH 8.0) as elution buffer. Protein concentration was determined by UV spectrophotometry at 280 nm, using the calculated molar absorptivity (21,890 M^−1^ cm^−1^) [29]. Additionally, the maximum absorbance peak was identified (474 nm) and the ratio of absorbance 474/280 was calculated (1.49) as control of purity.

#### YPDI and HuPDI purification

The pellet was resuspended in 5 volumes of lysis buffer (CelLytic^®^ B, as recommended by the manufacturer) and disrupted by rocking (15 min). Total cell lysate was clarified by centrifugation at 9,500 rpm for 15 min (4 °C). Recombinant YPDI or HuPDI were purified by Ni-chelating affinity chromatography following the native conditions protocol described in *the QIAexpressionist*^®^ *manual* (Qiagen). Finally, active PDI proteins were collected from Sephadex™ G-25 columns (PD-10, Amersham Biosciences) using 20 mM Tris-HCl (pH 8.0) as elution buffer. Protein concentration was determined by UV spectrophotometry at 280 nm, using the calculated molar absorptivity (M^−1^ cm^−1^: YPDI = 49,655; HuPDI = 45,755) [29]. The enzyme activity of PDI proteins was determined by an insulin-reduction turbidimetric assay reported elsewhere.

### 3.4. Acid-Denaturation of GFP

A 5 μM acid-denatured GFP solution (pH 1.5) was prepared by mixing a 10 μM GFP solution in denauring buffer (0.3 mM EDTA, 1 mM DTT, 50 mM Tris-HCl, pH 7.5) with an equal volume of 125 mM HCl solution and incubated at room temperature for 1 min [13]. After that, protein denaturation (low fluorescence signal) was confirmed by fluorescence measurements. Fluorescence was expressed in relative fluorescence units (RFU, *i.e.*, arbitrary florescence units per *p*mol of GFP).

### 3.5. Refolding of GFP by PDI Proteins

GFP refolding was started by diluting the 5 μM acid-denatured GFP in the renaturing buffer (25 mM MgCl_2_, 100 mM KCl, 50 mM Tris-HCl, pH 7.5) containing PDI enzymes. Refolding reactions were carried out in final volumes of 200 μL. All experiments were performed at room temperature and the fluorescence was continuously determined for 15 min. Fluorescence measurements were carried out using a *Fluoroskan Ascent*^®^ *FL* microplate fluorometer and luminometer (Thermo Scientific) at 485 nm excitation and 538 nm emission wavelengths.

### 3.6. Inhibition of the PDI Chaperone Activity by Bacitracin

The antibiotic bacitracin was used as inhibitor of the chaperone activity of PDI proteins. Different concentrations of bacitracin (0, 0.1, or 1 mM) were added to a renaturing buffer containing 2 μM of YPDI or HuPDI. After 30 min of the PDI/bacitracin interaction, the residual chaperone activity was measured as previously described, using 0.05 μM of acid-denatured GFP as substrate.

### 3.7. Statistical and Data Analysis

All statistical and data analysis were performed using Prism^®^ v.5 (GraphPad Software, San Diego, CA, USA). Mean, standard error of the mean (S.E.M.) and one-way ANOVA were calculated from data of three independent experiments. At a statistical value *p* < 0.05, the ANOVA was followed by either Tukey’s or Dunnett’s multiple comparison test, as appropriate. All acid-denatured GFP refolding data were fitted using the standard least square regression method. Association kinetics data were fitted to an exponential one-phase association model using the following Equation (1):

(1) $$Y=Y_{o}+(Plateau-Y_{o})(1-e^{-k\cdot t})$$

where *Y*_0_ and *Plateau* represent the fluorescence signal at time (*t*) equal to zero and at infinite time, respectively, while *k* is the observed constant rate of association. The chaperone concentration/*Plateau* data were fitted to a four-parameter logistic (4PL) dose-response variable slope model using the following Equation (2):

(2) $$Y=Y_{m}+\frac{Y_{M}-Y_{m}}{1+{\left(\frac{EC_{50}}{X}\right)}^{H}}$$

where *H* describes the steepness of the curves, *Y_m_* and *Y_M_* are the apparent plateaus of response, minimal and maximal respectively, and *EC*_50_ is the concentration of chaperone (*i.e.*, YPDI or HuPDI) that gives a response half way between *Y_m_* and *Y_M_*. The acid-denatured GFP concentration/*k* data were fitted to a specific one-binding site model using the following Equation

(3) $$Y=\frac{B_{max}\cdot X}{K_{d}+X}$$

where *B_max_* represents the maximum specific binding and *K_d_* is the equilibrium binding constant.

## 4. Conclusions

Eukaryotic PDI enzymes have been shown to be up-regulated during ER stress, suggesting that they might be involved in cellular protection, including protection against induced apoptosis [30,31]. Additionally, several studies have shown that PDI-mediated reductive cleavage of disulfide bonds at the cellular surface is essential for the infectivity of some pathogens, such as HIV, *Chlamydia*, and *Leishmania* [32–34]. Because of the diversity of their functional activities, PDI enzymes are potentially interesting drug targets. Given that PDI may be implicated in the protection of cells against ER stress, including cancer cells, inhibitors of PDI might be able to enhance the efficacy of chemotherapy in some cancers [35,36]; furthermore, it has been demonstrated that blocking the reductive cleavage of disulfide bonds of proteins associated with the cell surface markedly reduced the infectivity of HIV [37]. Although several high-throughput screening (HTS) assays to test PDI reductase activity have been reported [38,39], we described here a novel and simple micro-assay to test the chaperone activity of PDI enzymes that is amenable for HTS of PDI inhibitors.

## Figures and Tables

**Table 1:** Kinetic parameters of the chaperone activity exhibited by YPDI and HuPDI proteins using acid-denatured GFP as substrate*.

	Spontaneous	YPDI	HuPDI
*k* ( min^−1^)	0.037 ± 0.021	0.107 ± 0.012	0.311 ± 0.027
*Plateau* (RFU)	1.04 ± 0.48	1.91 ± 0.12	1.78 ± 0.04
EC_50_ (μM)	*n.d.*	0.45 ± 0.09	0.34 ± 0.04
*Hill* coefficient	*n.d.*	0.89 ± 0.11	1.06 ± 0.08
K_d_ (μM)	*n.d.*	0.30 ± 0.09	0.18 ± 0.03

* Data represent mean ± S.E.M. of three independent experiments; n.d., not determined.

**Table 2:** Primers used for the amplification and cloning of YPDI and HuPDI genes.

Primer	Sequence	Endonuclease
ScPDIF	acactc*ggatcc*CAACAAGAGGCTGTGGCC	*Bam*HI
ScPDIR	acactc*ctgcag*TTACAATTCATCGTGAATGGC	*Pst*I
BamPDIH	cg*ggatcc*GACGCCCCCGAGGAGGAGGAC	*Bam*HI
HsPDIR2	gccc*aagctt*ACAGTTCATCTTTCACAGCTTTCTG	*Hind*III
